# Association between MCP-1 -2518A>G polymorphism and asthma susceptibility: a meta-analysis

**DOI:** 10.1590/1414-431X20198549

**Published:** 2019-10-28

**Authors:** Wenli Chen, Jiewei Cui, Guoan Xiang, Jianpeng Zhang, Hongmei Gao

**Affiliations:** Respiratory Department, The Third Medical Center of Chinese People's Liberation Army General Hospital, Beijing, China

**Keywords:** MCP-1, Polymorphism, Asthma, Susceptibility, Meta-analysis

## Abstract

The published data on the association between MCP-1 -2518A>G polymorphism and asthma susceptibility are inconclusive. Therefore, we performed a meta-analysis to estimate the impact of MCP-1 -2518A>G polymorphism on asthma susceptibility. PubMed, Web of Science, Wanfang, and China National Knowledge Infrastructure (CNKI) databases were used to identify eligible studies. The pooled odds ratios (ORs) and corresponding 95% confidence intervals (CIs) were used to calculate the strength of association. Sensitivity analysis was performed to evaluate the influence of individual studies on the estimates of overall effect, and funnel plots and Egger's test were used to assess publication bias. Eight publications with 1562 asthma patients and 1574 controls were finally identified. Overall, we found no significant association between MCP-1 -2518A>G polymorphism and asthma susceptibility in any of the genetic model comparisons. After stratified analysis by ethnicity, the results showed that a significant association with asthma risk was found in Caucasians in all the genetic models. However, a protective association was found in Africans under the dominant model. The present meta-analysis suggested that the MCP-1 -2518 A>G polymorphism is a risk factor for asthma in the Caucasian population, nevertheless it has a protective effect in the African population.

## Introduction

Asthma is a chronic respiratory inflammation disease characterized by airway hyper-responsiveness, reversible airway obstruction, and airway wall remodeling to a variety of stimuli (1). Previous studies suggest that asthma is a multifactorial disease influenced by genetic and environmental factors (2,3). Several asthma susceptibility genes have been identified by the genome-wide association studies, of which the monocyte chemoattractant protein-1 (MCP-1, also termed as CCL2) has been extensively studied (4–6).

MCP-1, one of the CC chemokines families, appears to play a vital role in asthma pathogenesis because of its ability to attract monocytes and eosinophils, and activate mast cells and basophils, inducing leukotriene C-4 release into the airway, which directly induces airway hyper-responsiveness (7,8). MCP-1 also can drive undifferentiated T-lymphocyte populations towards IL-4-producing Th2-type cells, which is important in allergic inflammation (9). In addition, it has been demonstrated that neutralization of MCP-1 drastically reduces bronchial hyper-reactivity, lymphocyte-derived inflammatory mediators, and T-cell and eosinophil recruitment to the lung (10). Furthermore, elevated MCP-1 expression has been demonstrated in the bronchial epithelium of asthmatic patients (7), and comparison of the same asthmatic patients showed marked elevation of sputum MCP-1 levels preceding exacerbation of acute asthma attacks compared to the asymptomatic state (11). These accumulated data support the idea that MCP-1 plays an important role in asthma pathogenesis and the MCP-1 gene may be a susceptibility gene of asthma.

Up to now, a lot of genetic epidemiology studies have assessed the association of MCP-1 gene polymorphism and susceptibility of asthma in different populations. Most of them focused on MCP-1 -2518 A>G polymorphic site in the regulatory region. However, these results were inconclusive and inconsistent. Therefore, we performed a meta-analysis of all eligible studies to obtain a more precise estimation of the association of MCP-1 -2518 A>G polymorphism with asthma susceptibility.

## Material and Methods

This systematic review was conducted in accordance with the Preferred Reporting Items for Systematic Reviews and Meta-Analyses (PRISMA) Statement guidelines (<http://www.prisma-statement.org>).

### Publication search

Publications were searched using PubMed, Web of Science, Chinese National Knowledge Infrastructure (CNKI), and Wanfang databases (the last search was conducted on February 15, 2018). The search strategy utilized in our study was as follows: (asthma or asthmatic) and (MCP-1 or *CCL2*) in combination with (polymorphism or mutation or variant). Searching was performed in duplicate by two independent reviewers (WL Chen and JW Cui).

### Inclusion and exclusion criteria

The inclusion criteria of our study were as follows: 1) any human studies that estimated the prevalence of MCP-1 polymorphisms and asthma risk; 2) studies were published in English or Chinese; 3) genotype distributions or allele frequency of each study available for estimating an odds ratio (OR) with 95% confidence interval (CI); 4) sufficient results for data extraction, that is, number of subjects for each genotype in asthma and control groups. If eligible papers had insufficient information, we contacted authors by e-mail for additional information. Studies were excluded from our meta-analysis if they did not provide the related data.

### Data extraction

The basic information extracted for each study was as follows: name of first author, publication year, country, and ethnicity of cases and controls, age of cases, sample size, and genotype frequencies in cases and controls. Data were extracted independently and in duplicate by two reviewers (WL Chen and JW Cui) who used a standardized data extraction form. Any disagreement was adjudicated by a third author (HM Gao).

### Study quality assessment

Newcastle-Ottawa Scale (NOS) was used to assess the quality of the included studies. Items assessed included selection, comparability of cases and controls, exposure/outcome, age, and gender. The quality scores ranged from 0 to 9. We divided NOS score into three levels (higher quality, score ≥7; moderate quality, score 4 to 6; low quality, score <4).

### Statistical analysis

Hardy-Weinberg equilibrium was assessed for each study by the Pearson's chi-squared test in control groups. The OR with 95%CI was used to assess the strength of the association between MCP-1 polymorphism and asthma risk. The pooled OR for MCP-1 polymorphisms and asthma risk was calculated for dominant genetic model (AA+Aa *vs* aa), recessive genetic model (AA *vs* aa+Aa), homozygote genetic model (AA *vs* aa), and allele genetic model (A *vs* a). In this study, the aa genotype was a wild-type, while the AA genotype was a mutant. The Q-test and I^2^ test were used to assess the effect of heterogeneity. When the Q-test P value was >0.10 and I^2^ <50%, which indicates a lack of heterogeneity among studies, the fixed-effect model was used. Otherwise, the random effect model was used. Subgroup analysis was performed by ethnicity and age to further explore ethnicity-specific and age-specific effects. Sensitivity analysis was conducted by sequentially excluding one study at a time to examine the effect of each study on the combined result. Potential publication bias was investigated through a funnel plot. All statistical analyses were performed using the STATA 11.0 software (Stata Corp., USA).

## Results

### Characteristics of studies included in the meta-analysis

**Figure 1.**
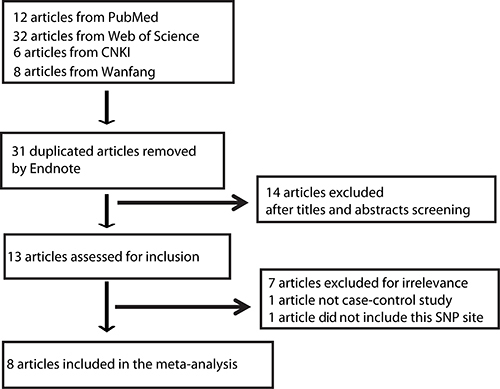
Flow diagram of inclusion of studies in the meta-analysis.

The flow diagram in Figure 1 outlines the study selection process. After a comprehensive search of databases, a total of 58 articles were identified. Firstly, we excluded 31 duplicated studies by Endnote software (Clarivate Analytics, USA). Then, 14 non-related articles were subsequently excluded after reading the titles and abstracts. The remaining 13 articles were further assessed for inclusion. Of these, 5 articles were excluded because 3 articles were irrelevant, 1 article lacked a case-control design, and 1 article focused on another MCP-1 SNP site. Eventually, we identified 8 case-control publications, including 1562 asthma patients and 1574 controls, to evaluate the association of MCP-1 -2518 A>G polymorphism with asthma susceptibility (12–19). There were three studies performed in Caucasian, three in Asian, and two in African populations. Six studies were carried out in children alone, one study in adults, and one in mixed. Polymorphisms in control subjects were in agreement with Hardy-Weinberg equilibrium (HWE) in all studies (P>0.05). The characteristics of each eligible study including the detailed genotype and allele frequencies and HWE examination are listed in Supplementary Table S1.

### Meta-analysis results

**Figure 2.**
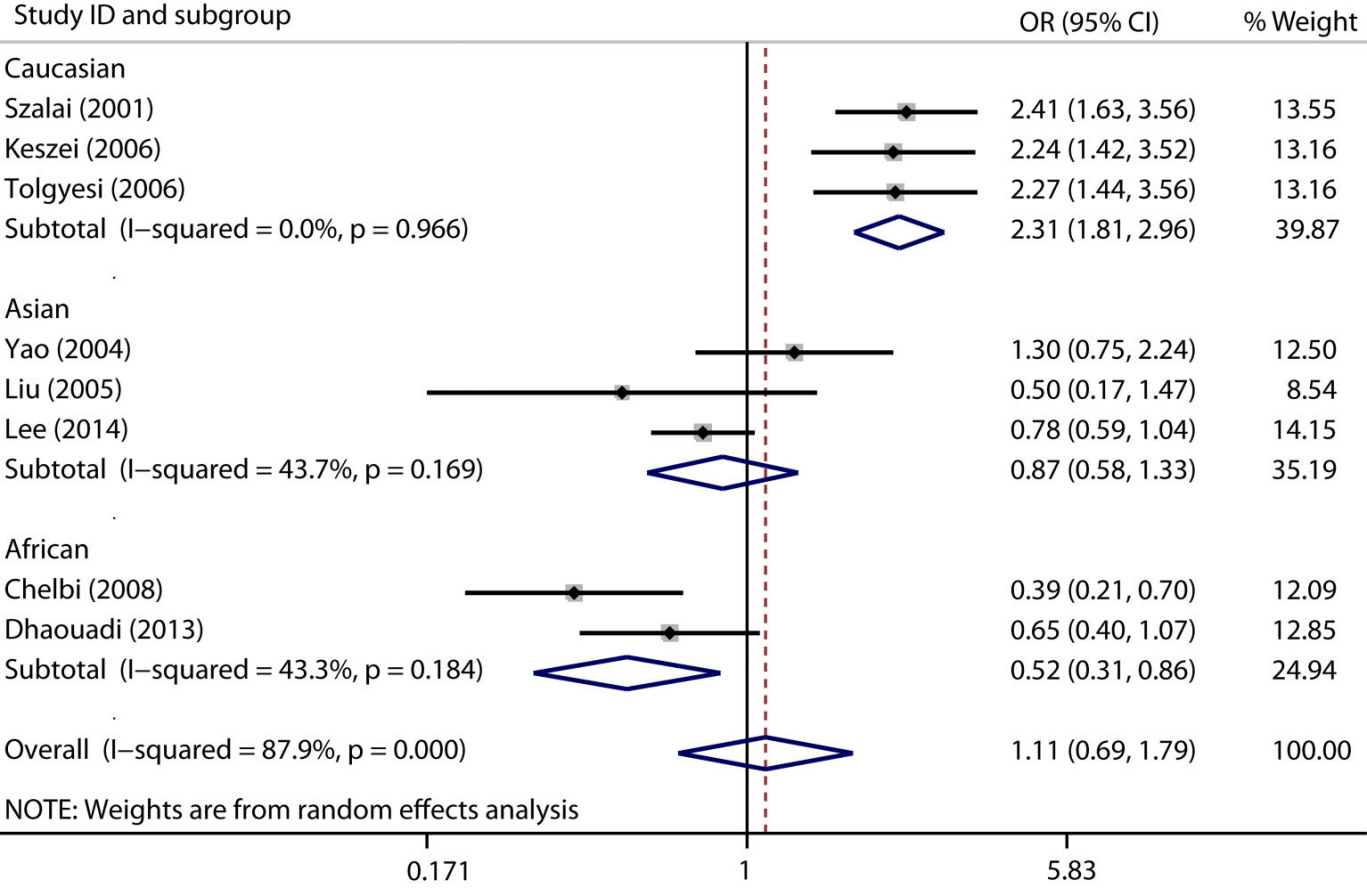
Forest plots of the association between the MCP-1 -2518 A>G polymorphism and risk of asthma in the dominant model.

A summary of the meta-analysis findings concerning association between MCP-1 -2518 A>G polymorphism and asthma susceptibility is provided in Figure 2 and Supplementary Table S2. Overall, no significant association was observed under the dominant model, recessive model, homozygote model, and allele model. After categorizing studies into different subgroups on the basis of ethnicity, the results showed that a significant association with asthma risk was found in the Caucasian population under the dominant model, recessive model, homozygote model, and allele model. However, a protective association was found in the African population under the dominant model. No significant association was found in the Asian population in any model. After categorizing studies into different subgroups on the basis of age, no significant association was found in the children subgroup in any genetic comparison model.

### Sensitivity and publication bias analysis

**Figure 3.**
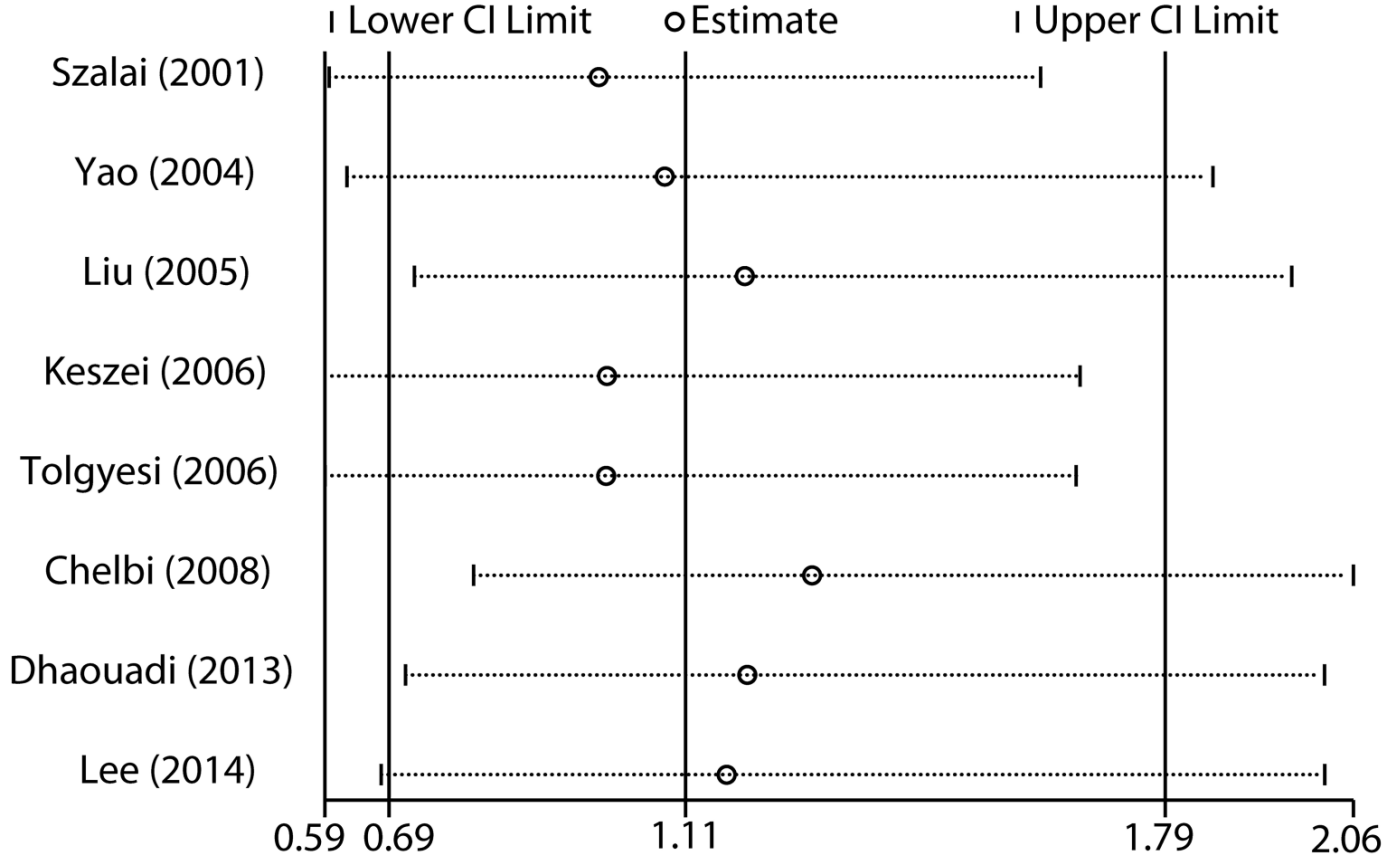
Sensitivity analysis for the MCP-1 -2518 A>G polymorphism and asthma susceptibility in the dominant model.

**Figure 4.**
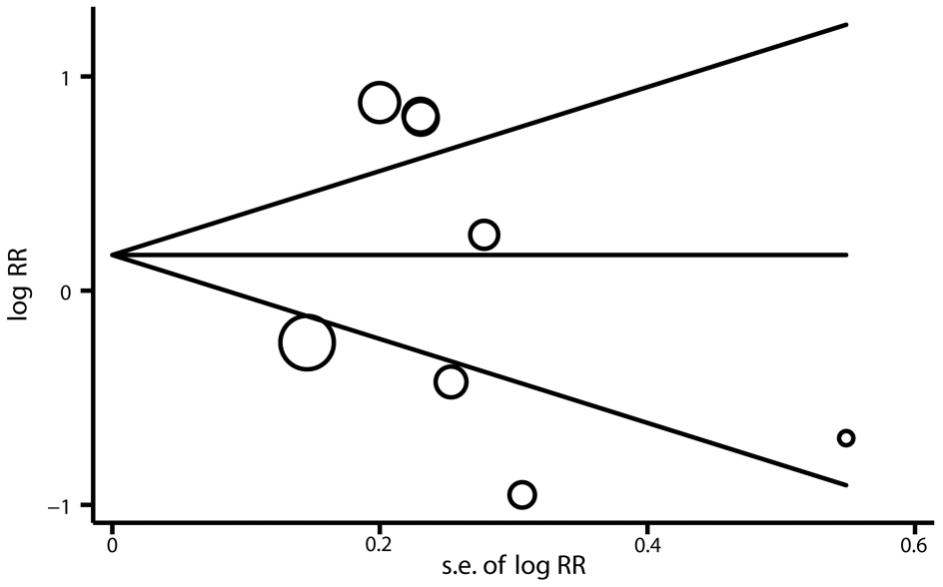
Begg's funnel plot for publication bias on MCP-1 -2518 A>G polymorphism and asthma susceptibility in the dominant model. s.e. indicates standard error.

Sensitivity analysis was conducted by sequentially excluding individual studies to estimate the stability of the results. After sequentially excluding each study, statistically similar results were found (Figure 3). Potential publication bias was investigated using a funnel plot. The shape of the funnel plot (Figure 4) did not indicate any evidence of publication bias for MCP-1 -2518 A>G polymorphism.

## Discussion

To the best of our knowledge, this is the first meta-analysis to assess the association between MCP-1 -2518 A>G gene polymorphism and asthma risk. Our results indicated that there was no significant effect of MCP-1 -2518 A>G polymorphism on asthma susceptibility in overall analyses. In the analysis stratified by ethnicity, an increased risk of asthma was seen with MCP-1 -2518 A>G polymorphism in the Caucasian population, however, a protective effect was seen in the African population.

The MCP-1 -2518 A>G polymorphism is located in the distal regulatory region of the MCP-1 gene (20). Functionally, this variant has been found to increase transcriptional activity of this region and subsequent MCP-1 production in response to an inflammatory stimulus (21). The -2518 A>G polymorphism has been reported to be associated with risk of rheumatoid arthritis (22) and lupus nephritis (23). Recently, an increasing number of studies explored the association of MCP-1 -2518 A>G polymorphism with susceptibility to asthma. The first study determining the relationship of MCP-1 -2518 A>G polymorphism and asthma was conducted by Szalai et al. (12). They found that the MCP-1 -2518 G polymorphism was correlated with asthma severity, and their results were subsequently supported by Keszei et al. (15) and Tolgyesi et al. (16). In contrast, two studies reported a protective effect of allele MCP-1 -2518 G polymorphism in asthmatic children (17,18), and three studies showed no significant association between MCP-1 -2518 G polymorphism and asthma risk (13,14,19). Our meta-analysis provided a more precise estimation based on a larger sample size compared with the individual study. The pooled results demonstrated that the MCP-1 -2518 G polymorphism was not associated with the susceptibility of asthma. The most possible explanation for these discrepancies among the different studies is due to the origins of the study populations. For instance, MCP-1 -2518 G allele frequency differs significantly in control samples from Tunisia, Hungary, and China ranging from about 51% in the Chinese population to 21% in the Hungarian population. Such discrepancies have also been observed for polymorphisms in the promoter region of another chemokine named CCL5 (or RANTES) (24–26). These reports reveal the ethnic diversity in allelic frequencies of these chemokine genes. In addition, a significant heterogeneity for MCP-1 -2518 A>G polymorphism and susceptibility of asthma among eight studies was observed in the meta-analysis. Interestingly, the heterogeneity was dramatically reduced or disappeared from the Caucasian subgroup after ethnicity subgroup analysis, and the subgroup pooled results showed an increased risk of asthma with MCP-1 -2518 G polymorphism in the Caucasian population, a protective effect for MCP-1 -2518 G polymorphism in the African population under the dominant model, and no significant association in the Asian population. Accordingly, the mean plasma MCP-1 protein level was significantly lower in the asthmatic than in the control children from Hungary (15), but plasma MCP-1 protein level was higher in the asthmatic than in the control children from Tunisia (17). The possible explanation is that the genetic variation in the MCP-1 promoter region not only affects the production of MCP-1, but also affects the secretion of MCP-1. However, the precise molecular mechanisms regulating MCP-1 secretion in individuals with genetic variations in the MCP-1 promoter region are currently not known.

There are some possible explanations for the different effects of MCP-1 -2518 G polymorphism on asthma between ethnicity. The mutant allele may have small but tightly linked effects in other possibly functional polymorphisms involved in the inflammatory response, which play more fundamental roles in asthma. Indeed, a study reported that the MCP-1 -2518 A>G polymorphism has strong linkage disequilibrium with other MCP gene polymorphisms, and the combination of MCP-1 -2518 A>G, MCP-2 +46A>C, and MCP-3 +563C>T was the best predictive model for asthma (19). In addition, there may be different inflammatory pathways involved in the pathogenesis of asthma.

Several limitations should be taken into account when interpreting our results. First, only published data were included, leading to possible publication bias in this meta-analysis, despite no statistically significant publication bias being identified. Second, even though the existing literature had acceptable quality, detailed information was not provided. Asthma definitions varied among different articles and this may be a confounding factor. In addition, participation rates for cases and controls were not reported in the majority of included studies, thus our meta-analysis was unable to explore the selection bias. Third, with limited information about maternal, constitutional, and environmental risk factors for asthma, the evaluation of the gene-gene and gene-environmental interactions was not possible.

In summary, the meta-analysis results indicated that the allele frequencies of MCP-1 -2518 A>G polymorphism were different among ethnicities, and the MCP-1 -2518 G polymorphism was a risk factor for asthma in the Caucasian population; nevertheless it had a protective effect in the African population. However, further large-scale studies are still required to confirm these results.

## Supplementary Material

Click here to view [pdf].
